# Plant Lectin, MoMo30, Pressures HIV-1 to Select for Variants with Deleted N-Linked Glycosylation Sites

**DOI:** 10.3390/v17070910

**Published:** 2025-06-27

**Authors:** Morgan I. Coleman, Mahfuz B. Khan, Erick Gbodossou, Amad Diop, Kenya DeBarros, Vincent C. Bond, Virginia Floyd, Kofi Kondwani, Valerie Montgomery Rice, Michael D. Powell

**Affiliations:** 1 Department of Microbiology, Biochemistry and Immunology, Morehouse School of Medicine, 720 Westview Dr. SW, Atlannta, GA 30310, USA; mcoleman@msm.edu (M.I.C.); mkhan@msm.edu (M.B.K.); kdebarros@msm.edu (K.D.); vbond@msm.edu (V.C.B.); 2 PROMETRA International, Dakar-Etoile BP 6134, Senegal; erick.gbodossou@gmail.com; 3 Malango Traditional Healers Association, Fatick BP 1763, Senegal; mikepowell5072@gmail.com; 4 Department of Community Health and Prevention, Morehouse School of Medicine, 720 Westview Dr. SW, Atlanta, GA 30310, USA; vfloyd@msm.edu (V.F.); kkondwani@msm.edu (K.K.); 5 Office of the President, Morehouse School of Medicine, 720 Westview Dr. SW, Atlanta, GA 30310, USA; vmrice@msm.edu

**Keywords:** HIV-1, HIV-1 genome MoMo30, drug-escalation study, plant lectin, CBA, gp120, HIV-1 envelope, *Momordica balsamina*

## Abstract

Momordica balsamina, a plant traditionally used in African medicine, contains a 30 kDa protein, MoMo30, previously identified by our group as an anti-HIV agent that binds glycan residues on the gp120 envelope protein, thereby acting as an entry inhibitor. In this study, we investigated whether prolonged exposure to MoMo30 exerts selective pressure on HIV-1 and induces mutations in the viral envelope (*env*) gene. T-lymphocyte cells were infected with HIV-1_NL4-3_ and continuously treated with MoMo30 over a 24-day period. Viral RNA was isolated at regular intervals, and *env* genes were sequenced using the Illumina platform. RNA sequence variant calling was performed using iVar, which uses a frequency-based binomial test with a default allele frequency threshold of 3% and a minimum base quality of 20 and applies Bonferroni correction for multiple testing. The infectivity of the MoMo30-exposed virus was assessed using MAGI-CXCR4 cells, visualized by β-galactosidase staining, and compared to untreated controls. Statistical significance was determined via two-way ANOVA. MoMo30-treated HIV-1 exhibited multiple detrimental mutations in gp120 and gp41, including missense, nonsense, and frameshift changes. Notably, 32% of N-linked glycosylation sites were deleted in the treated virus, while no such changes were observed in controls. Functionally, the MoMo30-treated virus demonstrated a sixfold reduction in infectivity compared to untreated HIV-1_NL4-3_. These findings suggest that MoMo30 imposes genetic pressure on HIV-1_NL4-3_, selecting for mutations that reduce viral fitness.

## 1. Introduction

Human immunodeficiency virus type 1 (HIV-1) is an enveloped retrovirus that primarily infects CD4^+^ T cells, macrophages, and dendritic cells, leading to progressive immune suppression and, if untreated, acquired immunodeficiency syndrome (AIDS). The viral life cycle begins when the envelope glycoprotein complex mediates attachment and fusion with the host cell membrane, facilitating entry and subsequent replication using host cellular machinery [1,2]. Although antiretroviral therapy (ART) has transformed HIV-1 infection into a manageable chronic condition, current treatments are not curative and require lifelong maintenance. ART targets several viral proteins—reverse transcriptase, integrase, protease, and envelope glycoproteins—to suppress viral replication and preserve immune function. Despite these advances, the development of a vaccine or a therapeutic capable of durably suppressing HIV-1 without continuous ART remains elusive [3,4,5,6].

One of the principal challenges in vaccine and drug development is the viral envelope glycoprotein, composed of gp120 and gp41, which mediates viral entry and plays a critical role in immune evasion. Gp120 is a heavily glycosylated surface protein that binds to the CD4 receptor on host cells, initiating entry [7,8,9]. It contains approximately 25–35 potential N-linked glycosylation sites (PNGSs), which vary between strains, and shields conserved epitopes from host immune recognition [10,11,12]. PNGSs are characterized by the consensus sequence N-X-S/T, in which N stands for asparagine, X is any amino acid except proline, and S/T is either serine or threonine. This sequence directs the enzymatic addition of a glycan chain to the asparagine residue, forming part of the viral glycan shield [10]. The glycan shield enables HIV-1 to evade neutralizing antibodies and reduces the efficacy of antiviral agents [13,14]. Following gp120 binding, gp41—a 41 kDa transmembrane protein non-covalently linked to gp120—facilitates membrane fusion, enabling viral entry. Due to their central roles in viral entry and immune evasion, gp120 and gp41 remain attractive yet challenging targets for HIV-1 therapeutics and vaccine design [8,15,16,17,18].

To overcome the obstacle posed by the glycan shield, recent research has focused on carbohydrate-binding agents (CBAs), which recognize and bind to specific glycan structures such as mannose or N-acetylglucosamine [19,20,21,22]. CBAs have shown promise as broad-spectrum antivirals that can bind to glycans on the viral envelope and block entry into susceptible cells. Plant lectins are a rich class of CBAs with anti-HIV-1 activity. Studies have shown that exposure to plant-derived CBAs such as *Hippeastrum* hybrid agglutinin (HHA), *Galanthus nivalis* agglutinin (GNA), and *Urtica dioica* agglutinin (UDA) can induce the deletion of N-linked glycans on gp120 and select for viral escape variants with reduced glycosylation. These glycan deletions may compromise viral fitness, offering a potential therapeutic advantage. Not only would glycan deletions destabilize the integrity of the envelope, but they may also expose previously hidden epitopes and illicit an innate immune response [23,24,25].

Our laboratory recently identified and characterized MoMo30, a novel CBA derived from *Momordica balsamina*, a plant used in traditional African medicine. MoMo30 is a 30 kDa plant lectin that acts as an entry inhibitor by binding to glycans on gp120, thereby blocking HIV-1 from entering target cells. We previously demonstrated that MoMo30 binds specifically to gp120 and prevents its interaction with T cells. In functional assays, MoMo30 inhibited both X4- and R5-tropic HIV-1 strains with greater potency than the FDA-approved entry inhibitor Enfuvirtide, achieving an IC_50_ of 2.8 nM against HIV-1_NL4-3_. MoMo30 also inhibited a diverse panel of primary HIV-1 isolates across different clades, with IC_50_ values ranging from 2.8 to 9.0 nM. Cytotoxicity assays indicated minimal toxicity, with CC_50_ values exceeding 33 µM in both MAGI and Jurkat cells, suggesting a favorable therapeutic index [26,27,28].

A distinctive feature of MoMo30 lies in its origin as a traditional medicinal remedy used by healers in West Africa for many years to manage HIV infection. Patients treated with extracts from *Momordica balsamina* have reported prolonged viral suppression following short-term treatment, a phenomenon that remains poorly understood scientifically. MoMo30 was sourced through a collaboration with PROMETRA International, an organization dedicated to preserving and promoting traditional medicine. This partnership has enabled us to investigate the active ingredient underlying the ethnomedicinal use of this plant and to characterize its antiviral mechanism in a rigorous laboratory setting.

Building upon this work, we sought to determine whether prolonged exposure to MoMo30 exerts selective pressure on HIV-1, resulting in mutations in the *env* gene and alterations in glycosylation patterns. Specifically, we aimed to (1) assess whether MoMo30 selects for variants with fewer N-linked glycosylation sites and (2) evaluate whether these changes affect viral infectivity. To date, few studies have comprehensively characterized the genotypic and phenotypic consequences of prolonged CBA exposure on HIV-1. Through this investigation, we aim to further elucidate the mechanism of action of MoMo30 and contribute to the growing body of literature exploring CBAs as promising therapeutic candidates for HIV-1.

## 2. Materials and Methods

### 2.1. HIV-1 Evolution Experiment with MoMo30 Treatment

We acquired the T-lymphocytic cell line Jurkat (clone E6-1) from the AIDS Reagent Program (NIH HIV Reagent Program, Germantown, MD, USA Cat # 177). We maintained them at 37 °C, with 5% CO_2_ in RPMI-1640 media (Fisher Scientific Company, Chicago, IL, USA, Cat # 10-040-CV) supplemented with 10% fetal bovine serum (FBS) (Med Supply Partners Atlanta, GA, USA, Cat # 62-1300-1) and 1X antibiotic–antimycotic (Fisher Scientific Company, Chicago, IL, USA, Cat # 5240-062).

To initiate the multi-round HIV-1 infection experiment, we infected two groups of Jurkat cells (5 × 10^6^ cells per condition in 1 mL of fresh RPMI-1640 medium supplemented with 10% FBS): one control group and one MoMo30-treated group. Both groups were infected with 150 ng p24 of HIV-1_NL4-3_. Our viral stock yields approximately 5000 blue cells per ng, which translates to an MOI of 0.15–0.2. The treated group also received 6 nM MoMo30 at the time of infection (Day 0), a concentration chosen to exert sufficient drug pressure (approximately 90–95% inhibition) based on the IC_50_ (~3 nM) determined from prior infection assays.

Infected cells were rotated on a nutator for 2 h at room temperature to facilitate viral adsorption. Afterward, they were centrifuged at 300× *g* for 5 min to remove the unbound virus, and the supernatant was discarded. Cells were washed twice with PBS (Fisher Scientific Company, Chicago, IL, USA, Cat# 14190-144) and then resuspended in 10 mL of fresh RPMI-1640 medium supplemented with 10% FBS. Since MoMo30 would have been removed during centrifugation and washing, 6 nM MoMo30 was re-added to the culture. After 24 h of incubation at 37 °C with 5% CO_2_, 10 mL of cell-free viral supernatant was collected and designated as Day 0.

**Figure 1:**
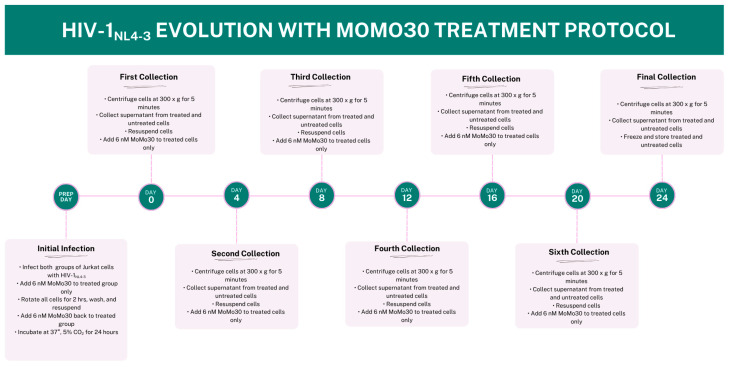
Timeline of HIV-1_NL4-3_ Evolution Experiment Under MoMo30 Treatment. Jurkat cells were infected with HIV-1_NL4-3_ (150 ng p24) and split into two groups: untreated (control) and MoMo30-treated (6 nM). Supernatants were collected every four days (Days 0, 4, 8, 12, 16, 20, and 24), with MoMo30 re-administered to treated cells after each collection through Day 20. On Day 24, the final supernatant was collected and frozen. This timeline outlines the sampling schedule and treatment workflow used to assess viral evolution and resistance under selective pressure.

Viral supernatants were collected every four days (Days 4, 8, 12, 16, 20, and 24). At each time point, cells were pelleted, and 10 mL of supernatant was harvested from both control and MoMo30-treated samples. Cells were then resuspended in 10 mL of fresh RPMI-1640 medium supplemented with 10% fetal bovine serum (FBS). For the MoMo30-treated group, 6 nM MoMo30 was re-added at each collection point through Day 20. No MoMo30 was added on Day 24, as this was the final collection. Figure 1 outlines the complete experimental timeline, from initial infection to final harvest.

To assess potential cytotoxic effects of MoMo30 independent of viral infection, two additional uninfected control groups were maintained under identical culture conditions for the full 24-day period: one containing untreated Jurkat cells (media only) and another treated with 6 nM MoMo30. These groups were monitored regularly via light microscopy. Cells in both groups displayed normal morphology and proliferation throughout the experiment, with no visible signs of cytotoxicity or growth inhibition.

### 2.2. Isolation of Virus from Supernatant

To isolate viral particles, 5 mL of culture supernatant from each sample was ultracentrifuged at 32,000× *g* for 2 h using a Beckman Coulter SW 40 Ti rotor (SN 7162) (Beckman Coulter, Inc. Indianapolis, IN, USA). Following centrifugation, the supernatant was carefully removed, and the viral pellets were resuspended in either phosphate-buffered saline (PBS) or lysis buffer, depending on the downstream assay. These concentrated viral preparations were then used to assess the impact of MoMo30 on multi-round HIV-1_NL4-3_ replication.

### 2.3. Total RNA Sequencing

For total RNA sequencing, samples from Days 0–12 and Days 13–24 were pooled into two composite samples. Viral RNA was extracted from viral particles using the QIAGEN RNeasy Mini Kit (Cat # 74104) (QIAGEN, Germantown, MD, USA) and sequenced on an Illumina platform by Genewiz^®^ (GENEWIZ from Azenta Life Sciences South Plainfield NJ, USA). Sequence data were processed using Galaxy, an open-source, web-based platform. Reads were trimmed to remove adapter sequences and low-quality bases and then aligned to HIV_NL4-3_ env86-561CpG, locus QGU22277.1; GenBank Accession: MN685350.1 using bwa v.0.7.12. Variant calling was performed with iVar v.1.3.1. to identify single-nucleotide polymorphisms (SNPs) and insertions/deletions (INS/DELs).

### 2.4. p24 ELISA

To quantify viral production over multiple rounds of infection, p24 levels in culture supernatants were measured using a commercial HIV-1 p24 ELISA kit (ImmunoDX, Cat. #103, Woburn, MA, USA) according to the manufacturer’s instructions. Test samples were diluted 1:5 in assay diluent and added to the plate. After a 1-h incubation at room temperature, plates were washed three times and incubated with detector reagent for another hour. Following a second wash, TMB substrate was added and incubated for 10 min. The reaction was stopped, and absorbance was measured at 450 nm using a microplate spectrophotometer (Agilent Technologies, Santa Clara, CA, USA).

### 2.5. Single-Round Infection Assay

U-373-MAGI-CXCR4CEM glioblastoma cells (NIH HIV Reagent Program Germantown, MD, USA, Cat # ARP-3596) were cultured to ~90% confluence and then infected with one ng p24-equivalent HIV-1_NL4-3_ viral supernatant derived from multi-round Jurkat cell infections. Infected cells were incubated at 37 °C with 5% CO_2_ for 48 h.

After incubation, cells were fixed by adding 1% formaldehyde (Fisher Scientific Company, Chicago, IL, USA, Cat # F-79–500) and 0.2% glutaraldehyde (Fisher Scientific Company, Chicago, IL, USA, Cat # F-02957-1) in PBS directly to each well. To detect infected cells, a β-galactosidase staining solution was prepared by combining 14.25 mL of PBS, 300 µL of 0.2 M potassium ferrocyanide, 300 µL of 0.2 M potassium ferricyanide, 15 µL of 2 M MgCl_2_, and 150 µL of 40 mg/mL X-gal in DMSO. Two milliliters of this solution were added to each well, and the plates were incubated at 37 °C for 50 min. Afterward, cells were washed with PBS, and blue-stained (infected) cells were visualized and counted using light microscopy (Olympus BX53, Olympus Corporation, Waltham, MA, USA).

### 2.6. Statistical Analysis

A two-way ANOVA was used for the p24 detection ELISA and the single-round infection assay, with a *p* < 0.05 as statistically significant. RNA sequence variant calling was performed using iVar. iVar employs a frequency-based binomial test, utilizing a default allele frequency threshold of 3% and a minimum base quality of 20, and applies the Bonferroni correction for multiple testing. Variants that do not meet the threshold are excluded.

## 3. Results

### 3.1. RNA Sequencing

To assess the genotypic effects of MoMo30 on the HIV-1_NL4-3_ envelope proteins gp120 and gp41 following multiple rounds of replication under treatment pressure, we performed RNA sequencing using the Illumina platform. Viral RNA was extracted from supernatants collected every 4 days over a 24-day period. Samples from Days 0–12 and Days 13–24 were pooled and sequenced to identify the number, type, and distribution of mutations within the *env* gene. Sequencing quality metrics, including read count, average read length, depth of coverage, and base quality, are summarized in Table 1.

### 3.2. Analysis of Potential N-Linked Glycosylation Site Mutations

Potential N-linked glycosylation sites (PNGSs), defined by the consensus motif N-X-S/T (where N = asparagine, X = any residue except proline, and S/T = serine or threonine), serve as attachment points for glycan chains and contribute to the viral glycan shield. To assess whether MoMo30 treatment alters envelope glycosylation, we analyzed changes in the number and distribution of PNGSs across the entire *env* gene, which can dynamically shift, emerge, or disappear under selective pressure.

In the control virus, the number of PNGSs remained constant throughout the experiment (31 sites), showing no gain or loss during either the early (Days 0–12) or late (Days 13–24) phases. Similarly, MoMo30-treated samples retained all 31 PNGSs during the early phase. However, in the late phase, the treated virus lost 10 PNGSs, representing a 32% reduction relative to the reference genome. This reduction suggests that MoMo30 exerts selective pressure that promotes the loss of glycosylation sites, potentially altering envelope structure or immune evasion strategies.

A summary of these glycosylation site changes by condition and time point is shown in Table 2.

### 3.3. Analysis of gp120 Variable Loop Mutationsa

To assess the impact of MoMo30 on the genetic integrity of the HIV-1 *env* gene, we analyzed amino acid changes within the gp120 variable loops (V1–V5) over the 24-day treatment period. In the untreated control virus, no substitutions were observed in these regions during either the early (Days 0–12) or late (Days 13–24) phases, indicating genetic stability in the absence of selective pressure.

In contrast, the MoMo30-treated virus exhibited no changes in the variable loops during the early phase (Days 0–12). However, in the late phase (Days 13–24), we detected significant mutational activity within the gp120 variable regions. These included amino acid substitutions, frameshifts, and the introduction of premature start and stop codons, affecting a total of 25 residues. While mutations were observed across multiple variable loops (V1/V2, V3, V4, and V5), the majority were concentrated in the V3 loop. Notably, a mutation at position Pro264 introduced a start codon, while a mutation at Arg280 generated a premature stop codon—both of which could severely disrupt gp120 structure and function. Several mutations were located within regions previously associated with viral tropism, immune recognition, or envelope stability. For example, substitutions at Arg276 and Gly277 within the V3 loop may alter local charge or structural flexibility. The mutation Arg280 → Stop is expected to truncate gp120, and Pro264 → Start may reflect a shift in the translational start site. The presence of multiple amino acid substitutions at a single position (e.g., Asn265 → Tyr/Asp/Ile) suggests the emergence of diverse viral quasi-species in response to the treatment pressure of MoMo30. A summary of the amino acid changes in gp120 variable loops and their potential impact on gp120 is shown in Table 3.

### 3.4. Analysis of Mutations with gp41 Functional Domains

gp41 contains highly conserved domains essential for the membrane fusion process required for viral entry, including the N-terminal heptad repeat (HR1), the C-terminal heptad repeat (HR2), the homotrimer interface, and the membrane-proximal external region (MPER). Mutations in these regions can impair fusion efficiency, reduce viral replication, or diminish infectivity.

Throughout the 24-day experiment, the control virus showed no amino acid changes in any gp41 domain. Likewise, during the early phase of MoMo30 treatment (Days 0–12), no amino acid-altering mutations were observed. However, in the late phase (Days 13–24), the MoMo30-treated virus accumulated numerous amino acid changes across gp41, including substitutions, frameshifts, and the gain of start and premature stop codons. Most of these changes were localized to HR1, HR2, and MPER. Notably, a start codon emerged at Ser115, and premature stop codons were introduced at Leu112, Glu119, and Ser120—sites within HR2 and adjacent regions. Several positions exhibited multiple variants, consistent with the emergence of diverse viral quasi-species under selective pressure.

Table 4 summarizes the most functionally relevant gp41 mutations observed following MoMo30 treatment. A full list of detected mutations is provided in Supplementary Figure S1.

### 3.5. p24 LEISA: Effects of MoMo30 on HIV Replication over Time

To monitor HIV-1_NL4-3_ replication, we quantified p24 capsid antigen levels in culture supernatants from MoMo30-treated and untreated Jurkat cells over a 24-day period using a standard p24 ELISA. p24 is a core structural protein of HIV-1 and a well-established marker of viral replication, as its concentration correlates with the production and release of viral particles.

From Day 0 to Day 8, p24 concentrations were similar across both groups, indicating equivalent levels of initial infection and early viral replication. By Day 12, divergent trends emerged. In untreated cultures, p24 levels continued to rise, peaking at Day 20, followed by a transient decline and rebound by Day 24. This is a biphasic pattern typical of multi-round HIV-1 replication in vitro.

In contrast, MoMo30-treated samples plateaued between Days 8 and 12 and subsequently declined. By Day 24, p24 levels had dropped to ~10 ng/mL, less than half of that in untreated cultures, indicating sustained suppression of viral replication.

A two-way ANOVA confirmed significant effects of treatment (F(1,14) = 138.7, *p* < 0.0001), time (F(6,14) = 179.3, *p* < 0.0001), and their interaction (F(6,14) = 25.45, *p* < 0.0001). Most of the variation (77.89%) was attributed to time, with treatment and interaction effects contributing 10.04% and 11.06%, respectively.

Importantly, the observed decline in p24 is unlikely due to cytotoxic effects. Our prior study demonstrated that MoMo30 is non-toxic to Jurkat cells at concentrations below 1 mg/mL, with cytotoxicity only observed at concentrations of 30,000 nM or higher. In the present study, only 6 nM was used, supporting a direct antiviral mechanism rather than cell death [27] (Coleman et al., Int J Environ Res Public Health, 2022).

**Figure 2:**
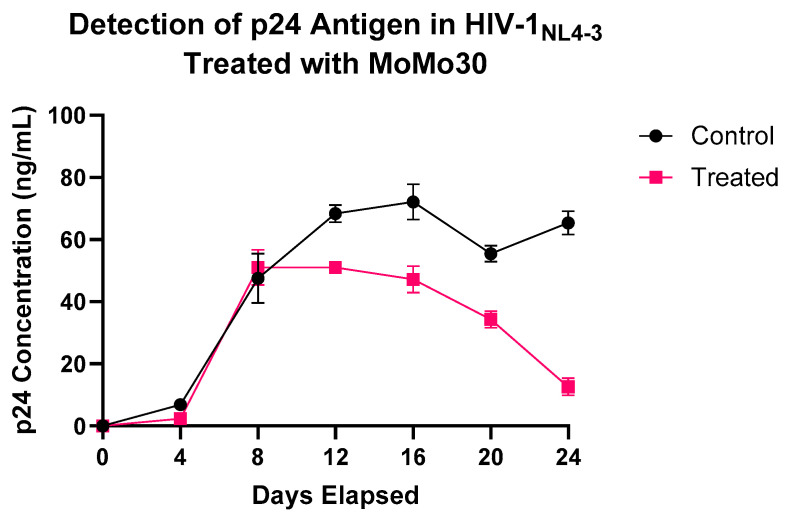
MoMo30 suppresses HIV-1_NL4-3_ replication in Jurkat cells over time. p24 antigen levels were measured by ELISA in culture supernatants collected every four days from MoMo30-treated and untreated HIV-1_NL4-3_-infected Jurkat cells. Untreated cultures continuously produced increasing amounts of viruses, peaking at Day 20. MoMo30-treated cultures showed a steady decline in p24 levels after Day 12. Data represents standard deviations of triplicate measurements. A two-way ANOVA confirmed significant effects of treatment, time, and their interaction (*p* < 0.0001 for all comparisons).

These results indicate that MoMo30 effectively attenuates sustained HIV-1_NL4-3_ replication over time. Figure 2 depicts the trend of p24 concentration over time.

### 3.6. Single-Round Infection Assay

To evaluate the effect of MoMo30 on HIV-1_NL4-3_ infectivity, we performed a MAGI infectivity assay using virus collected from treated and untreated cultures across multiple time points. In untreated samples, infectivity was high by Day 4, with more than 2500 infected cells detected. This level remained relatively stable through Day 24, with a modest increase from Days 4 to 16, a slight dip on Day 20, and a rebound by Day 24.

In contrast, MoMo30-treated virus showed markedly reduced infectivity at all time points. Virus collected on Day 4 failed to infect any cells, and samples from Days 8 and 12 yielded fewer than 500 infected cells, representing an approximately sixfold reduction compared to the untreated virus. By Day 16, infectivity was completely abolished in the treated group, and this loss persisted through Day 24.

A two-way ANOVA confirmed significant effects of treatment (F(1,14) = 13,206, *p* < 0.0001), time (F(6,14) = 506.1, *p* < 0.0001), and their interaction (F(6,14) = 400.4, *p* < 0.0001). Treatment accounted for 70.78% of the total variance, while time and the interaction contributed 16.27% and 12.88%, respectively. The mean number of infected cells across all time points was 2470 in the untreated group and 151 in the MoMo30-treated group, representing a mean difference of 2319 cells.

**Figure 3:**
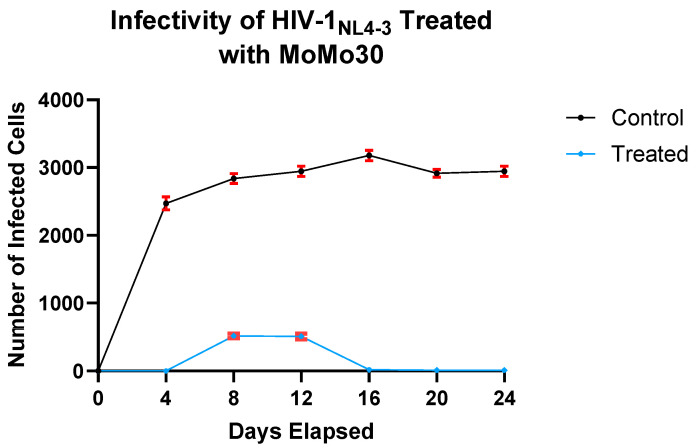
MoMo30 reduces HIV-1_NL4-3_ infectivity in Jurkat cells over time. Virus collected from untreated cultures showed high and sustained infectivity over the 24-day period. In contrast, the virus from MoMo30-treated cultures exhibited a sharp decline in infectivity over time, with near-complete loss by Day 16. Data represents the means ± standard deviations of triplicate wells. A two-way ANOVA confirmed significant effects of treatment, time, and their interaction (*p* < 0.0001 for all comparisons), with MoMo30 treatment accounting for over 70% of the observed variance.

These data demonstrate a substantial, time-dependent reduction in HIV-1_NL4-3_ infectivity following MoMo30 treatment (Figure 3).

## 4. Discussion

Our study demonstrates that exposure to MoMo30 imposes selective pressure on HIV-1_NL4-3_, leading to the deletion of approximately 32% of potential N-linked glycosylation sites (PNGSs) and a concurrent decline in viral infectivity. The glycosylation of HIV-1 envelope glycoproteins is essential for immune evasion, receptor binding, and structural stability [9,29,30]. The loss of PNGSs can unmask conserved epitopes and destabilize the envelope structure, rendering the virus more vulnerable to immune detection and less capable of efficient replication [31,32].

We analyzed mutations across the envelope gene to gain a deeper understanding of the molecular basis for this reduced infectivity. Several mutations occurred in functional domains of gp41, including the heptad repeats (HR1 and HR2) and the membrane-proximal external region (MPER), which are typically conserved due to their essential role in membrane fusion and viral entry [8,33]. Frameshifts and nonsense mutations in these domains likely disrupt the six-helix bundle required for fusion. Additionally, numerous mutations were identified in the variable loops of the gp120 protein. The variable loops are involved in coreceptor binding and epitope shielding. Although these loops tolerate some variability, excessive mutation can alter loop conformation and length, compromising receptor engagement and impairing entry [34,35,36,37,38]. Notably, the deleted PNGSs were not reconstituted elsewhere in the *env* gene, despite the virus’s high mutation rate. This suggests a selective viral strategy to avoid MoMo30 recognition even at the cost of envelope integrity [39,40,41].

Our in vitro results further support this mechanism. In MAGI infectivity assays, untreated HIV-1 initiated robust infection by day 4 (~2500 infected cells), while MoMo30-treated virus showed no detectable infection until day 8, with only approximately 500 infected cells, representing an 80% reduction. However, p24 antigen levels in both groups remained comparable through day 8, indicating that viral particle production was not initially inhibited. This disconnect between the viral load and infectivity implies that MoMo30 initially acts by rendering virions non-infectious rather than blocking replication. Likely, MoMo30 binds to glycan moieties on gp120 and gp41, preventing the conformational changes necessary for receptor binding or fusion pore formation. This interference may delay viral entry, prolong the eclipse phase, and reduce overall infectivity without impairing post-entry replication.

These findings position MoMo30 within a promising class of carbohydrate-binding agents (CBAs) that target conserved glycan structures on enveloped viruses, including HIV-1, influenza, Ebola, and SARS-CoV-2 [21,25,42,43,44,45]. Unlike traditional antiretrovirals, CBAs physically block viral entry by binding glycan epitopes on envelope glycoproteins. MoMo30’s broad glycan specificity, particularly for mannose and N-acetylglucosamine [26], enables binding across diverse viral strains. This broad recognition may raise concerns about off-target effects, including host glycoprotein binding and potential immunogenicity. However, viral glycan density, structure, and spatial presentation differ significantly from those on host cells [46,47,48]. CBAs, such as MoMo30, likely exploit these differences to preferentially bind viral glycans over host glycans. Moreover, CBAs can be engineered to reduce nonspecific binding, improve pharmacologic stability, and minimize immune activation, thereby enhancing their therapeutic potential [21,49,50].

Although HIV-1 can evolve under selective pressure, our findings suggest that resistance to MoMo30 comes at a high fitness cost. The sustained loss of PNGSs and structural mutations in gp120 and gp41 reflect a viral strategy to evade glycan-mediated MoMo30 binding. Yet, these adaptations destabilize the envelope and expose conserved epitopes, increasing immune visibility. Thus, the virus faces a functional impasse: retaining glycosylation sites promotes immune evasion but enhances MoMo30 susceptibility, whereas deleting them reduces MoMo30 binding but exposes the virus to host defenses. In essence, the virus is trapped; any adaptation that enables it to escape one pressure heightens its vulnerability to another.

From a therapeutic perspective, the forced glycan deletion induced by MoMo30 may inadvertently prime the immune system. By unmasking conserved regions typically hidden by the glycan shield, MoMo30 could facilitate the generation of broadly neutralizing antibodies (bNAbs). This immunomodulatory effect resembles a “self-vaccination” phenomenon, wherein the host mounts adaptive responses against structurally weakened viral variants. Notably, reports from traditional healer communities describe durable viral suppression following short-term administration of *Momordica balsamina*, suggesting a potential novel mechanism of sustained antiviral activity. Understanding these effects will be critical for evaluating MoMo30’s full potential, not only as a direct antiviral agent but also as a candidate for vaccine development or adjunct immunotherapy [51,52,53,54,55,56,57].

While our findings provide valuable insights into the antiviral activity of MoMo30, this study is not without limitations. Future studies will incorporate primary HIV-1 isolates and primary human cells to assess whether MoMo30 elicits comparable antiviral effects under more physiologically relevant conditions. Additionally, while we observed a substantial loss of N-linked glycosylation sites in the MoMo30-resistant virus, the specific contributions of individual PNGS mutations to resistance remain to be dissected. Functional studies are needed to determine whether particular glycan deletions confer differential effects on MoMo30 binding, viral fitness, or immune recognition. Furthermore, a critical next step will be to characterize the host immune response following MoMo30 treatment. In particular, profiling the B-cell repertoire and evaluating the breadth and potency of neutralizing antibody responses will be essential to determine MoMo30’s potential not only as a direct antiviral agent but also as an immunogenic compound. Together, these efforts will clarify MoMo30’s utility in therapeutic and vaccine development settings.

## 5. Conclusions

In this study, we investigated the impact of MoMo30, a novel antiviral plant lectin, on the envelope glycoprotein of HIV-1_NL4-3_. Our findings demonstrate that MoMo30 exerts selective pressure on the virus, leading to the emergence of mutant variants with alterations in critical regions of the *env* gene. Three key conclusions emerged from our analysis. First, mutations accumulated over time: no changes were observed during the early phase of treatment, and multiple mutations emerged during the later phase, indicating time-dependent selection. Second, prolonged exposure to MoMo30 led to deletions in potential N-linked glycosylation sites (PNGSs), resulting in the loss of 32% of PNGSs and multiple mutations within conserved regions of gp41, which are essential for viral fusion and infectivity. Third, MoMo30-induced mutations were associated with a substantial reduction in viral infectivity. While replication dynamics, as measured by p24 antigen levels, remained similar between treated and untreated viruses through the first 8 days, the MoMo30-treated virus displayed a profound delay and reduction in infectivity. In MAGI infectivity assays, untreated virus robustly infected cells by day 4 (~2500 infected cells), whereas treated virus showed no infection by day 4 and only ~500 infected cells by day 8—a sixfold reduction. This disconnect between viral replication and infectivity strongly suggests that MoMo30 does not initially inhibit viral replication but rather interferes with viral entry and/or membrane fusion by disrupting the functional conformation of the envelope glycoprotein.

Together, these findings suggest that MoMo30 exerts antiviral pressure, driving HIV-1 toward variants with compromised envelope function and reduced infectivity. By selectively targeting viral glycan structures, MoMo30 impairs HIV-1 entry while preserving virion production, offering a unique mechanism of action distinct from traditional antiretrovirals. These results support the further development of MoMo30 as a promising entry inhibitor and potential immunomodulator capable of exposing conserved epitopes to the host immune system.

## 6. Patents

This work is covered by the US patents US20220054601A1, US11925670B2, US 11,266,723 B1, and US 11,414,462.

## Figures and Tables

**Table 1:** Sequencing statistics for HIV-1_NL4-3_ samples collected during MoMo30 treatment. Summary of high-throughput sequencing metrics for viral RNA samples collected from untreated and MoMo30-treated HIV-1_NL4-3_ cultures during early (Days 0–12) and late (Days 13–24) phases. Metrics include total read count, average read length, estimated depth of coverage (based on a 9173 bp genome), and average quality per read (Q ≥ 39), confirming high-confidence variant detection across all samples.

Sequencing Statistics per Sample				
Sample	Read Count	Average Read Length	Depth of Coverage (x)	Average Quality per Read
Untreated HIV-1_NL4-3_ Days 0–12	1,798,483	150	~29,411	≥39
Untreated HIV-1_NL4-3_ Days 13–24	4,693,741	150	~76,736	≥39
MoMo30 Treated HIV-1_NL4-3_ Days 0–12	2,461,268	150	~40,247	≥39
MoMo30 Treated HIV-1_NL4-3_ Days 13–24	3,692,770	150	~60,391	≥39

**Table 2:** Loss of potential N-linked glycosylation sites (PNGSs) in the HIV-1_NL4-3_ envelope gene following MoMo30 treatment. This chart illustrates the number of potential N-linked glycosylation sites (PNGSs) in the HIV-1_NL4-3_ *env* gene (accession MN685350.1) during the experiment. The MoMo30-treated virus exhibited a 32% reduction in PNGSs, with 10 sites permanently deleted by Days 13–24. In contrast, the untreated control virus retained a stable PNGS count throughout the experiment.

Changes in Potential N-Linked Glycosylation Sites (PNGSs) in HIV-1_NL4-3_ Envelope Gene			
Condition	# of PNGSs Days 0–12	# of PNGSs Days 13–24	Change from Reference
Untreated HIV-1_NL4-3_	31	31	No change
MoMo30 Treated HIV-1_NL4-3_	31	21	Decrease of 10 PNGSs during Days 13–24

**Table 3:** Summary of the location, type, and potential impact of each mutation relative to the reference HIV-1_NL4-3_. This table summarizes amino acid changes observed in the gp120 variable loops of the HIV-1_NL4-3_ envelope gene during a MoMo30 treatment experiment. No mutations were detected in the untreated (control) virus at either the early or late time points, nor in the MoMo30-treated virus at the early time point. However, multiple mutations were observed in the MoMo30-treated virus at the late time point, including single-nucleotide polymorphisms (SNPs), insertions, and deletions. Several of the SNPs introduced novel start or stop codons, potentially altering protein structure or function.

Amino Acid Changes in gp120 Variable Loops After MoMo30 Treatment		
Variable Loop	Amino Acid Changes After MoMo30 Treatment	Potential Impact
V1	No changes observed	—
V2	- Arg263→Pro - Asn265→Tyr/Asp/Ile - Asn267→Leu - Thr268→Pro	- Loss of positive charge and flexibility (Arg→Pro) - Asn265 mutations may disrupt glycosylation site - Asn267→Leu removes potential glycan and adds hydrophobicity - Proline at 268 may introduce kink in backbone
V3	- Gln275→Val/Gly - Arg276→Phe/Leu - Gly277→Leu/Ser - Pro278→Ser	- Shift from polar to hydrophobic (Gln→Val) - Loss of positive charge and introduction of bulky hydrophobic residues (Arg276) - Possible structural rigidity increase (Gly→Leu/Ser) - Pro→Ser substitution may reduce loop constraint
V4	- Gln293_Ala294 ins12 - Ala294→Asp - His295→Leu	- Insertion likely alters local structure/loop length - Ala→Asp introduces negative charge - His→Leu shifts from polar to hydrophobic, may affect receptor interactions
V5	- Arg280→Stop codon - Asp439→Glu	- Premature stop likely truncates gp120, disrupting structure - Asp→Glu is conservative but may alter local charge density

**Table 4:** Detailed summary of mutations in gp41 functional domains after MoMo30 treatment. Detailed breakdown of amino acid substitutions, insertions, and stop codons detected in the late phase of MoMo30-treated virus. Mutations cluster in key regions of gp41, including the homotrimer interface region, core helices, and MPER residues, suggesting selection pressure and possible functional impact.

Key Amino Acid Changes in gp41 Functional Domains After MoMo30 Treatment		
Primary Domain	Amino Acid Changes After MoMo30 Treatment	Potential Impact
N-TERMINAL HEPTAD REPEAT (HR1)	- Arg28→Ser - Ala32→Cys/Ser - Gln33→Leu	- May disrupt coiled-coil heptad repeat; may affect bundle formation - Introduction of cysteine may lead to aberrant disulfide bonding - Hydrophobic substitution may alter helical interface
HOMOTRIMER INTERFACE (HR1 LOOP)	- Ala78→Ser - Val79→Glu - Trp94→Cys	- Polar substitution may affect interface packing - Negative charge could disrupt hydrophobic core - Loss of bulky aromatic residue; may impair trimer stability
C-TERMINAL HEPTAD REPEAT (HR2)	- Leu112→Stop - Ser115→Val - Glu119→Stop	- Premature truncation; likely disrupts HR2 function - Hydrophobic substitution may affect helical stability - Early stop codon likely abolishes six-helix bundle formation
MEMBRANE PROXIMAL EXTERNAL REGION (MPER)	- Glu121→Cys - Trp137→Ser - Trp144→Leu	- Cysteine introduction may affect folding or disulfide linkage - Loss of aromatic residue; may reduce membrane interaction - Hydrophobic substitution; could impair MPER-mediated fusion

## Data Availability

The original contributions presented in this study are included in the article/Supplementary Material. Further inquiries can be directed to the corresponding author.

## Supplementary Materials

The following supporting information can be downloaded at: https://www.mdpi.com/article/10.3390/v17070910/s1, Figure S1. Full list of amino acid changes in gp41 functional domains. Comprehensive summary of amino acid substitutions, insertions, and stop codons identified during the late phase of MoMo30-treated virus. Mutations are concentrated in critical regions of gp41, including the homo-trimer interface, heptad repeats 1 and 2 (HR1 and HR2), and membrane-proximal external region (MPER). These changes indicate selective pressure and potential functional consequences.
