# Supplementary material for: Plant Lectin, MoMo30, Pressures HIV-1 to Select for Variants with Deleted N-Linked Glycosylation Sites
Source: Viruses. 2025 Jun 27;17(7):910. doi: 10.3390/v17070910 (PMC12300216; doi:10.3390/v17070910)
Supplement: Supplementary file 1 [file viruses-17-00910-s001.zip › viruses-3727151-supplementary.pdf]

Supplemental Material

S1. All Amino Acid Changes in gp41 Functional Domains After MoMo30 Treatment

Functional Domains	Amino Acid Changes	Potential Impact
**Homotrimer Interface, HR1**	- Val10_Gln11→Ser - Gln11→Ser - Ala12→Ser - Arg13_Gln14 ins4 - Gln14_Leu15 ins5 - Leu15_Leu16 ins4 - Leu16_Ser17 ins2 - Ile19_Val20 ins4 - Val20_Gln21 ins13 - Leu26→Ser - Arg28→Ser - Ile30_Glu31 ins3 - Glu31_Ala32 ins4 - Ala32→Cys/Ser - Gln33→Leu - Gln34_His35 ins5	- Clustered insertions and substitutions may disrupt alpha-helical packing and destabilize trimer interface - Cysteine addition (Ala32→Cys) may introduce aberrant disulfide bonding
**Homotrimer Interface**	- Ile66→Thr - Ala78→Ser - Val79→Glu - Trp94→Cys	- Polar/charged residue substitutions may weaken trimer integrity - Trp94→Cys introduces thiol group, potentially affecting structure or antibody recognition
**Homotrimer Interface, HR2**	- Leu112→Stop - Ile113_His114 ins13 - Ser115→Val - Glu119→Stop - Ser120→Stop	- Premature stop codons truncate gp41, likely abrogating function - Mutations in HR2 may impair six-helix bundle formation and membrane fusion
**HR2, MPER**	- Glu121→Cys - Gln123_Gln124 ins3 - Gln124_Glu125 ins3 - Glu125_Lys126 ins2 - Asn145_Ile146 ins18 - Ile146_Thr147 ins2 - Asn148_Trp149 ins15	- Cysteine at position 121 may cause improper folding - Insertions may lengthen loop regions and interfere with MPER-mediated fusion or immune recognition
**MPER**	- Gln130→Gln (synonymous) - Glu133→Leu - Trp137→Ser - Phe144→Tyr - Trp144→Leu	- Trp and Phe substitutions could reduce MPER anchoring or antibody binding affinity - Mixed hydrophobic/aromatic changes may impact Env membrane interactions

Figure S1. Full list of amino acid changes in gp41 functional domains. Comprehensive summary of amino acid substitutions, insertions, and stop codons identified during the late phase of MoMo30-treated virus. Mutations are concentrated in critical regions of gp41, including the homo-trimer interface, heptad repeats 1 and 2 (HR1 and HR2), and membrane-proximal external region (MPER). These changes indicate selective pressure and potential functional consequences.
